# Clinical Randomized Controlled Study of Acupuncture Treatment on Children with Autism Spectrum Disorder (ASD): A Systematic Review and Meta-Analysis

**DOI:** 10.1155/2021/5549849

**Published:** 2021-07-24

**Authors:** Lei Wang, Jin-Lin Peng, Fu-Qiang Qiao, Wen-Ming Cheng, Guang-Wen Lin, Yao Zhang, Tian-Ge Gao, Ying-Ying Sun, Wei-Zhong Tang, Pu Wang

**Affiliations:** ^1^Department of Rehabilitation Medicine, The Seventh Affiliated Hospital, Sun Yat-Sen University, Shenzhen 518107, Guangdong, China; ^2^Tongji Hospital, Tongji Medical College, Huazhong University of Science & Technology, Wuhan 430000, Hubei, China; ^3^School of Education and Psychology, University of Jinan, Jinan 250000, Shandong, China; ^4^Department of Wei Zhong Children's Rehabilitation Center, Jinan 250000, Shandong, China

## Abstract

This study aimed to summarize the effectiveness and safety of acupuncture in the treatment of autism spectrum disorder (ASD) through literature analysis and evaluation. All studies were retrieved from various databases as follows: English databases, such as PubMed, Cochrane Library, Ovid, and Web of Science, and Chinese databases, such as China National Knowledge Infrastructure (CNKI), WanFang Data (WF), and Technology Periodical Database (VIP). The Cochrane Collaboration's Bias Risk Assessment Scale was used to assess the studies' risk of bias. The effects of acupuncture treatment for ASD were determined using the following indicators: childhood autism rating scale (CARS), autism behavior check list (ABC), Reynell developmental language scale (RDLS), and functional independence measure of children (WeeFIM). The risk map of bias of these studies' quality and the meta-analysis results of the indicators was prepared with RevMan 5.2 software. Finally, 16 studies were included, five of which were in English and 11 were in Chinese. The 16 studies included 1332 patients. The CARS results for subgroup analysis were as follows: acupuncture subgroup (MD = −2.65, 95% CI (−3.22, −2.07)) and acupuncture plus massage subgroup (MD = −10.35, 95% CI (−11.34, −9.36)). The ABC results were as follows: (MD = −6.70, 95% CI (−9.10, −4.29)). The analysis results of sensory, relating, language, body and object use, and social/self-help in the subitems of ABC were as follows: sensory (MD = −2.67, 95% CI (−2.90, −2.44)), relating (MD = −3.28, 95% CI (−3.55, −3.02)), language (MD = −2.45, 95% CI (−2.73, −2.16)), body and object use (MD = −1.19, 95% CI (−1.38, −1.00)), and social/self-help (MD = −2.09, 95% CI (−2.30, −1.89)). For the analysis results of comprehension and expression ages in the subitems of RDLS, the comprehension age results were as follows: (MD = 0.08, 95% CI (−0.06, 0.22), *P* = 0.27). Those of expression age were as follows: (MD = 0.15, 95% CI (0.04, 0.26), *P*=0.009). The WeeFIM results were as follows: (MD = 3.70, 95% CI (2.38, 5.02)). This study suggested that acupuncture could effectively treat ASD. However, acupuncture methods and prescriptions at this stage remain heterogeneous, and acupuncture treatment operations require standardization. Studies using rigorous and standard research designs are needed to draw stronger conclusions about the advantages of using acupuncture to treat children and adolescents with ASD.

## 1. Introduction

Autism spectrum disorder (ASD), also known as autism, is a type of mental development disorder that starts before the age of 3 years old. ASD is characterized by social communication disorders, limitations, stereotypes, and repetitive behaviors. ASD is now categorized as a mental illness [1, 2]. In epidemiological surveys, the median global prevalence of ASD was 62 in 10,000 [3]. According to the Second Epidemiological Sample Survey of the Disabled, in China, approximately 41,000 children aged 0–6 years old have ASD [1]. According to the report of the World Health Organization (WHO) in 2017, approximately 1 in 160 children suffers from ASD, and this proportion is still increasing [4]. ASD is accompanied by various problems, such as abnormal emotions, abnormal behaviors, and developmental disorders. Children with ASD usually incur long-term social and family medical costs. In the United States, the estimated total annual cost of children with ASD is between $11.5 billion and $60.9 billion [5]. The medical expenses of children and adolescents with ASD are 4.1–6.2 times those of children and adolescents without ASD [6].

Improving the curative effect of treatments of patients with ASD has become a long-term concern of the clinical medical staff. Nowadays, the commonly used clinical interventions for ASD include behavioral interventions and drugs. Behavioral interventions are more common than drugs [7]. However, behavioral interventions require large-scale venues and specialized facilities, one-to-one treatment, and the help of professionals trained in institutions for long-term work; these requirements consume human resources and are expensive [8]. The effectiveness of behavioral interventions is limited [9], and it is usually combined with other approaches, such as drugs [10]. Drug treatment also has limitations. The current drugs used for treating ASD are only helpful for a small number of people. The US Food and Drug Administration (FDA) has approved very few drugs for the treatment of ASD [11]. Drugs serve only to reduce irritability and hyperactivity behavior and are not effective in reducing social and language barriers for most patients with ASD [12]. In addition, long-term drug use results in side effects, such as weight gain, fatigue, drowsiness, and tremors, for example, risperidone treatment [13, 14].

Considering the abovementioned reasons, researchers are focusing more on the role of complementary and alternative medicine in ASD. In the United States, complementary and alternative medicines used to treat ASD involve diet modifications and food supplements [10]. Acupuncture is rarely used as an intervention for children with ASD in the United States [15]. However, in China and other Asian countries, acupuncture is widely used in the treatment with ASD. In a survey conducted in Hong Kong, acupuncture has reportedly been used on approximately 40% of children with ASD; it has become the most common form of complementary and alternative medicine used for this condition [16]. In China, acupuncture and other TCM methods are considered alternative therapies that need to be verified in accordance with the guidelines for the diagnosis, treatment, and rehabilitation of children with autism issued by the Ministry of Health in 2010 [1]. The treatment of ASD with acupuncture can be performed in many ways, including electroacupuncture [17], tongue acupuncture [18, 19], scalp acupuncture [20, 21], and total body acupuncture [10].

In this study, a meta-analysis of clinical randomised controlled studies was performed by searching relevant articles. The aims of this study were as follows: to evaluate acupuncture's efficacy and safety in the treatment of children and adolescents with ASD, to further search for stronger evidence that supports the use of acupuncture as a treatment for ASD, and to analyze the deficiencies of current studies.

## 2. Methods

### 2.1. Study Search

All studies were retrieved from various databases as follows: English databases, such as PubMed, Cochrane Library, Ovid, and Web of Science, and Chinese databases, such as China National Knowledge infrastructure (CNKI), WanFang Data (WF), and Technology Periodical Database (VIP). Articles published from the establishment of the database to October 2020 were included in the search. The articles must either be in Chinese or in English. We performed several presearches based on related subject words and free words. We determined the final search formula and collected articles based on this formula. The search terms were as follows: acupuncture, scalp acupuncture, electroacupuncture, tongue acupuncture, traditional Chinese medicine (TCM), autism spectrum disorders, ASDs, ASD, and autism. Two independent authors (Lei Wang and Jin-lin Peng) conducted a literature search according to the search strategy. The disputed part was addressed by a third author (Pu Wang).

### 2.2. Inclusion and Exclusion Criteria

The inclusion criteria were as follows: (a) participants were diagnosed with ASD, (b) the intervention method involved an experimental group subjected to acupuncture, and (c) the study type was a randomised controlled one. The exclusion criteria were as follows: (a) the conventional treatment in the intervention measures did not meet the treatment recommended by the guidelines, (b) reviews and repeated articles, (c) the full text being unavailable or articles having incomplete data, and (d) animal studies.

### 2.3. Data Extraction

We extracted the following data: (a) normal information including first author, year of publication, sample size, acupoint, age, gender, intervention measures, and treatment course; (b) outcome indicators, including childhood autism rating scale (CARS), autism behavior check list (ABC), subitems of ABC, Reynell developmental language scale (RDLS), subitems of RDLS, and functional independence measure of children (WeeFIM). Two independent authors (Lei Wang and Jin-lin Peng) conducted a literature search according to the search strategy. The disputed part was addressed by a third author (Pu Wang).

### 2.4. Quality Evaluation

The included studies' quality was evaluated by two independent authors (Lei Wang and Jin-lin Peng). If a disagreement arose during the review process, a third author made the decision. The Cochrane Collaboration's Bias Risk Assessment Scale was used to assess the studies' risk of bias. Each study was assessed for selection bias (random sequence generation and allocation concealment), performance bias (blinding of participants and personnel), detection bias (blinding of outcome assessment), attrition bias (incomplete outcome data reporting), and reporting bias (selective outcome reporting). Each domain was rated as follows: high risk of bias, unclear bias, or low risk of bias [22]. A risk map of bias of these studies' quality was prepared by using RevMan 5.2 software. GRADEpro GDT online tool was used to assess the quality of evidence. The tool is available at the official website: http://www.guidelinedevelopment.org/.

### 2.5. Statistical Analysis

The Review Manager 5.2 software of Cochrane Collaboration was used for the meta-analysis. The outcome variables were continuous. Thus, the mean difference (MD) was calculated for the results, and the 95% CI of the statistical results was reported. The chi-square test was used to calculate the heterogeneity of the included literature. When heterogeneity was *P* > 0.1 and *I*^2^ < 50%, a fixed-effect model was used. When the heterogeneity was *I*^2^ > 50%, the causes of heterogeneity were analyzed through a subgroup or sensitivity analysis. If the results still had heterogeneity, then the random-effect mode was used for summary analysis [22]. If the number of included studies was sufficient (*n* ≥ 10), then the funnel chart was used to perform a bias analysis.

## 3. Results

### 3.1. Search Results

**Figure 1:**
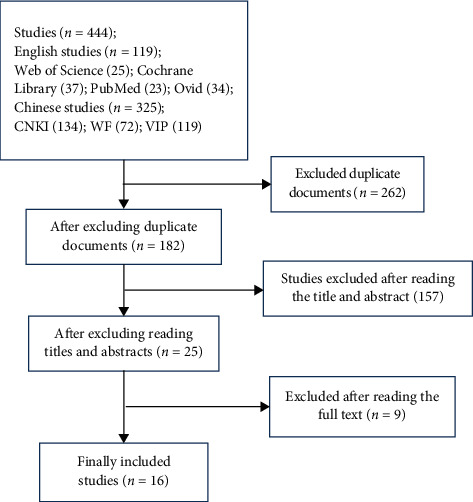
Flow chart of the study selection process.

A total of 444 studies were retrieved, and all were imported into the Document Management Software of “Medical Literature King V6.” A total of 182 duplicate studies were eliminated by using the function of duplication removal, 157 studies were excluded after reading the title and abstract, and 9 studies were excluded after reading the original text. Finally, 16 studies were included [17–21, 23–33], as shown in Figure 1.

### 3.2. Risk of Bias

**Figure 2:**
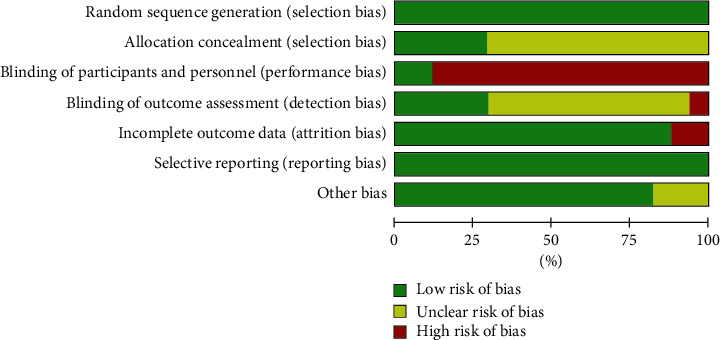
Risk of bias in included studies (A).

**Figure 3:**
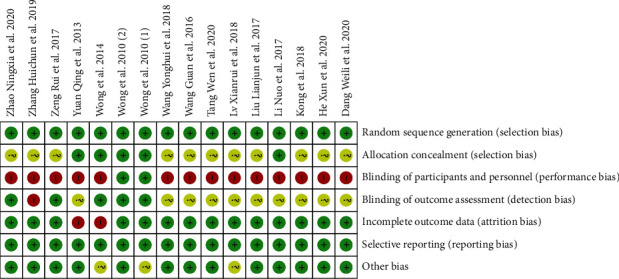
Risk of bias in included studies (B).

The results of Cochrane Collaborative Network Bias Risk Assessment Scale Evaluation are shown in Figures 2 and 3. Twelve studies reported the source of random sequences, and five explained the implementation of allocation-hiding schemes [17–19, 30, 33]. Guaranteeing the blinding method in acupuncture and rehabilitation measures was difficult. Thus, only two studies were included to ensure the double-blinding of the experiment. The two groups were provided control groups to implement the method of sham acupuncture for double-blinding [17, 19]. Five studies reported that the evaluation of experimental results was blinded [17–19, 26, 29], whereas one study reported that the evaluation of the outcome was not blinded [21]. Two studies had insufficient data integrity and did not report and analyze the data on patients lost to follow-up and those who withdrew from the study [18, 30].

### 3.3. Quality of Evidence

The study included a total of 12 outcome indicators. The GRADEpro GDT online tool was used to assess the quality of evidence. Only the acupuncture subgroup of CARS showed moderate quality. CARS showed low quality, and the other indicators showed very low quality. The results are shown in Table 1.

### 3.4. Study Characteristics

As shown in Table 2, the characteristics included in the studies were as follows: first author, year of publication, sample size, acupoint, age, gender, intervention measures, and treatment course.

### 3.5. Outcome Analysis

#### 3.5.1. CARS Analysis

**Figure 4:**
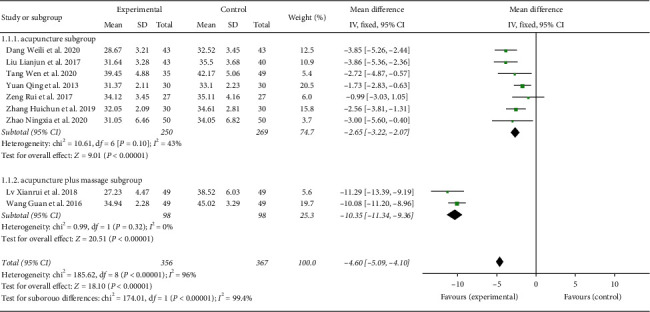
Forest plot showing MD (with 95% CI) for CARS of included studies comparing the experimental group with the control group. Childhood autism rating scale (CARS).

A total of 723 participants were included in nine studies [21, 23–26, 28–31], *I*^2^ = 96%. Heterogeneity existed. We performed a subgroup analysis. According to the intervention factors of the experimental group, the group was divided into two subgroups, namely, acupuncture and acupuncture plus massage [23, 28]. For the acupuncture subgroup, *P*=0.10 and *I*^2^ = 43%. Thus, we selected the fixed-effect model (MD = −2.65, 95% CI (−3.22, −2.07), (*P* < 0.00001)). For the acupuncture plus massage subgroup, *P*=0.32 and *I*^2^ = 0%. Thus, we used a fixed-effect model (MD = −10.35, 95% CI (−11.34, −9.36) (*P* < 0.00001)). The analysis results of both subgroups were statistically significant, as shown in Figure 4.

#### 3.5.2. ABC Analysis

**Figure 5:**
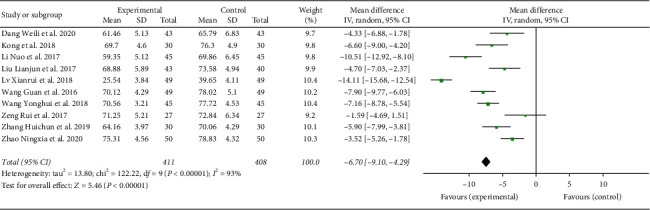
Forest plot showing MD (with 95% CI) for ABC of included studies comparing the experimental group with the control group. ABC: autism behavior checklist.

A total of 819 participants were included in 10 studies [17, 21, 23, 25, 26, 28, 29, 31–33]. Results showed that heterogeneity (*I*^2^ = 93%) existed. Through subgroup and sensitivity analyses, no significant change in heterogeneity was found. We selected the random-effect model (MD = −6.70, 95% CI (−9.10, −4.29), *P* < 0.00001). The difference between the two groups was found to be statistically significant, as shown in Figure 5.

#### 3.5.3. Analysis of ABC's Subitems

**Figure 6:**
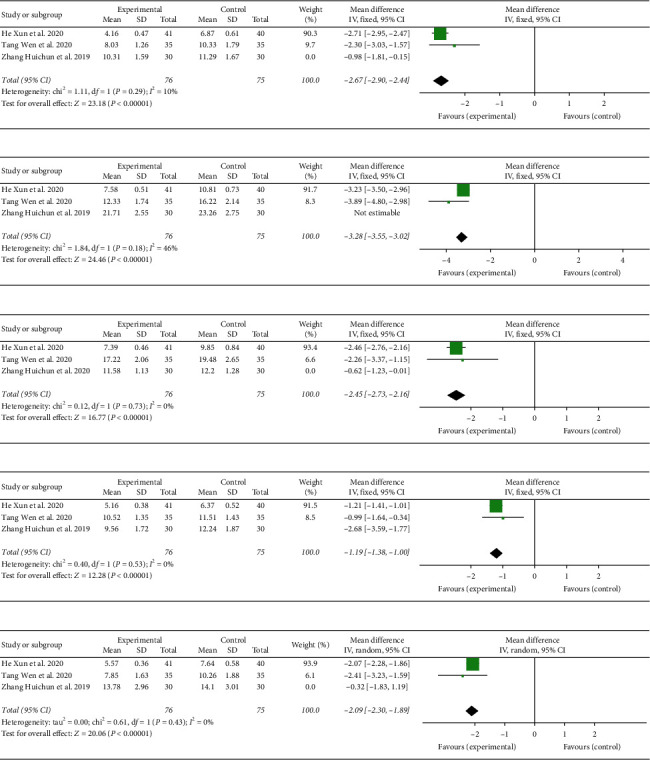
Forest plot showing MD (with 95% CI) for subitems of autism behavior check list (ABC) of included studies comparing the experimental group with the control group. ABC, subitems of ABC included sensory, relating, language, body and object use, and social/self-help. (a) Sensory, (b) relating, (c) language, (d) body and object use, and (e) social/self-help.

A total of 211 participants were included in three studies [21–24, 27]. The analysis results of sensory, relating, language, body and object use, and social/self-help in the subitems of ABC showed heterogeneity as follows: *I*^2^ = 88%, *I*^2^ = 75%, *I*^2^ = 93%, *I*^2^ = 83%, and *I*^2^ = 66%, respectively. Further sensitivity analysis revealed that the control group in literature (Zhang Huichun, 2019) used the Taiwanese Kidd children's sensory integration training, which differs from two other studies in terms of conventional rehabilitation training [21]. This factor was analyzed as a possible cause of heterogeneity, and the analysis was performed after removing it. As shown in Figure 6, the fixed-effect model results were as follows: sensory (*P*=0.29, *I*^2^ = 10%), (MD = −2.67, 95% CI (−2.90, −2.44), *P* < 0.00001) (Figure 6(a)); relating (*P*=0.18; *I*^2^ = 46%), (MD = −3.28, 95% CI (−3.55, −3.02), *P* < 0.00001) (Figure 6(b)); language (*P*=0.73, *I*^2^ = 0%), (MD = −2.45, 95% CI (−2.73, −2.16), *P* < 0.00001) (Figure 6(c)); body and object use (*P*=0.53; *I*^2^ = 0%), (MD = −1.19, 95% CI (−1.38, −1.00), *P* < 0.00001) (Figure 6(d)); and social/self-help (*P*=0.43; *I*^2^ = 0%), (MD = −2.09, 95% CI (−2.30, −1.89), *P* < 0.00001) (Figure 6(e)).

#### 3.5.4. RDLS Analysis

**Figure 7:**
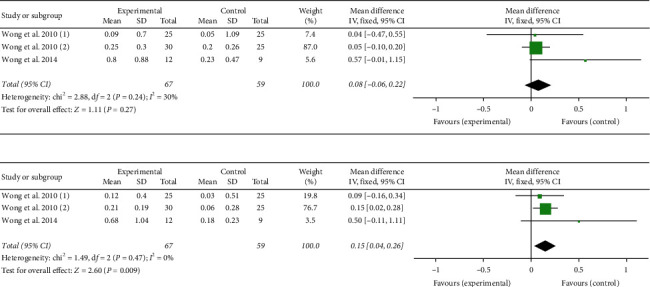
Forest plot showing MD (with 95% CI) for subitems of Reynell developmental language scale (RDLS) of included studies comparing the experimental group with the control group. Subitems of RDLS included comprehension and expression ages. (a) Comprehension age; (b) expression age.

A total of 126 participants were included in three studies [17–19].  Figure 7 presents the analysis results of comprehension and expression ages in the subitems of RDLS. The results for comprehension age were as follows: (*P*=0.24; *I*^2^ = 30%), select fixed-effect model, (MD = 0.08, 95% CI (−0.06, 0.22), *P*=0.27), and the two groups were statistically significant (Figure 7(a)). The results for expression age were as follows: (*P*=0.47; *I*^2^ = 0%), select the fixed-effect model, (MD = 0.15, 95% CI (0.04, 0.26), *P*=0.009), and the two groups were statistically significant (Figure 7(b)).

#### 3.5.5. WeeFIM Analysis

**Figure 8:**
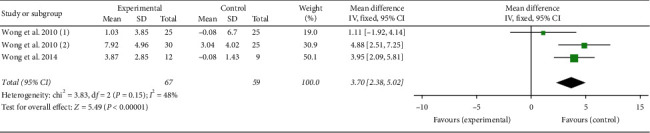
Forest plot showing MD (with 95% CI) for WeeFIM of included studies comparing the experimental group with the control group. WeeFIM: functional independence measure of children.

A total of 126 participants were included in three studies [17–19]. The analysis results were as follows: (*P*=0.15; *I*^2^ = 48%), select fixed-effect model, (MD = 3.70, 95% CI (2.38, 5.02), *P* < 0.00001), and the two groups were statistically significant (Figure 8).

#### 3.5.6. Publication Bias

**Figure 9:**
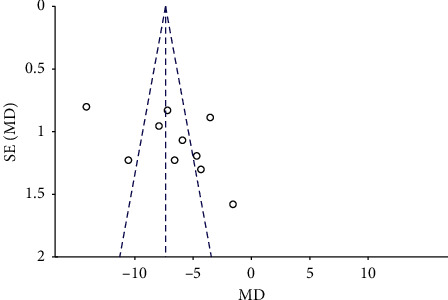
Funnel graph for ABC of included studies. ABC: autism behavior checklist.

Among the observed outcome indicators, only ABC included 10 studies, and the other outcome indicators included in the study were <10. A funnel chart analysis was performed on ABC.  Figure 9 shows that the distribution of the funnel graph was asymmetric, and a certain degree of bias was present. The following problems existed in the analysis of the literature: the sample size of the included studies was generally low, the risk of publication bias in small randomised controlled trials may be high, and the included literature was only in Chinese and English, thereby excluding the literature in other languages. All of the above factors may cause publication deviation, as described in detail in Table 2.

#### 3.5.7. Safety

Only four studies [17–19, 25] reported the safety of acupuncture for the treatment of ASD. A study [25] reported the presence of a subcutaneous bruise at the puncture site. The symptoms were mild and did not affect the treatment course. The remaining three studies [17–19] reported that some children experienced minor superficial bleeding or cried and showed irritability during acupuncture. The other included studies did not report safety issues.

## 4. Discussion

This review included 16 studies, which included 1332 patients. The experimental group had statistical differences in the scores of CARS, RDLS, WeeFIM, and ABC, and the subitems were compared with the control group. Among the 16 studies, two studies [17, 19] compared acupuncture and sham acupuncture. Results of analysis confirmed the effectiveness of acupuncture in the treatment of ADS. The other studies on the two groups' intervention methods compared rehabilitation treatment and rehabilitation treatment plus acupuncture. Results suggested that the combination of acupuncture with other treatments improved the treatment effect. The findings suggested that acupuncture alleviated the various symptoms of ASD.

Acupuncture differs from the modern medicine theory, which states that autism is caused by brain dysfunction. In TCM, autism is believed to be mostly caused by congenital insufficiency, which leads to an imbalance in body function. The cause lies in the brain and is closely related to the heart, liver, and kidneys [34]. Symptoms can be alleviated by applying acupuncture to relevant acupoints, that is, stimulating the head acupoints, namely, Si shencong (EX-HN1), Shenting (GV 24), Benshen (GB 13), Yintang (GV 29), Naohu (GV 17), and Naokong (GB 19), as well as other acupoints that can alleviate ASD. These acupoints are mostly distributed in the projection area of the frontal, parietal, and temporal lobes on the body surface. Stimulating these acupoints can adjust cortical function and brain electrical activity, thereby improving cerebral blood flow speed, promoting functional awakening and recovery, and improving intelligence. Acupuncture can alleviate affective disorder, attention disorder, and abnormal behavior [26]. Shenmen (HT 7) is one of the most commonly used acupoints in the acupuncture treatment of ASD. Wang Yu et al. found that acupuncture at Shenmen can regulate neurotransmitters, promote the secretion of neurotrophic factors, regulate the nerve-endocrine-immune network system, and inhibit cell apoptosis, thereby improving brain function [35]. Tongue acupuncture is one of the acupuncture methods [18, 19]. According to TCM, the tongue is the intersection site of all 14 meridians. The physiological mechanism might be related to the fact that the tongue is close to the brainstem and cerebellum; thus, stimulating tongue acupoints might augment the neural pathways connected to the motor-somatosensory cortex [17]. In a randomised controlled trial that used a positron emission tomography scan of the brain as one of the outcome measures for acupuncture versus no acupuncture, results showed that acupuncture alleviated some core features of ASD [17].

Sixteen studies were finally included, and all of them reported the source of random assignment sequence. Only five studies [17–19, 30, 33] described allocation hiding. The remaining studies did not provide specific descriptions. Given that acupuncture and rehabilitation cannot guarantee blindness, only two studies [17, 19] implemented double blindness, and both groups were assigned a control group that was treated with sham acupuncture. Five studies [17–19, 26, 29] reported blinding in the evaluation of experimental results. One study [21] reported that the outcome evaluation was not blinded, whereas the remaining studies did not specify it. In summary, a certain risk of bias exists in the study-quality evaluation results. Results suggested that clinical trials and reports are insufficiently clear and precise.

In conclusion, acupuncture was found to be effective and safe in the treatment of ASD. However, the use of GRADEpro GDT online tool to classify the quality of evidence for the study indicated that the quality of most outcome indicators were low and very low. Only the acupuncture subgroup of CARS showed moderate quality. The conclusion of the study was very likely to be very different from the real situation. The most degraded factor was the limitation of the study, which showed that the randomised controlled trials included in the study had a large bias in method design, as reflected in the insufficient or unreasonable implementation of blinding and allocation concealment. In addition, the study also had high risks in terms of heterogeneity, accuracy, and publication bias.

In the literature, the acupuncture types and methods were relatively diverse. According to the form of acupuncture, some studies used medical staff to stimulate acupuncture points through manual manipulation [25, 36]. Some studies used electric current to stimulate acupuncture points; electrical devices were used to perform acupuncture [17, 37]. Acupuncture was classified according to the body part treated as follows: body acupuncture [17], head acupuncture [31], latch acupuncture [18, 19], and so on. Additionally, under the guidance of the TCM theory, different acupuncture points were selected based on changes in different symptoms and syndrome types. Existing research shows no standardized and unified treatment plan for the acupuncture treatment of autism, and the treatment prescription required further standardization.

In the included literature, only one study [17] reported the results of follow-up and patient compliance. Four studies [17–19, 25] reported side effects. No obvious side effects were reported in the results. A study by Dang Weili et al. [25] reported a subcutaneous bruise at the puncture site. The symptoms were mild and did not affect the treatment course. The remaining three studies [17–19] reported that some of the children experienced minor superficial bleeding and cried or showed irritability during acupuncture. Most children easily adapted and tolerated this technology. The compliance of children with autism is a problem in the treatment process, and for acupuncture method, the compliance of children is expected to be difficult. However, most of the included studies did not report compliance problems and low-compliance solutions for children.

In summary, acupuncture exerted a certain curative effect on ASD and could alleviate ASD's core symptoms. However, acupuncture methods and prescriptions at this stage remain heterogeneous. Large sample size, rigorous research, and long observation and follow-up periods are required to prove the advantages of acupuncture's curative effect on ASD. In addition, studies need to use rigorous and standard research designs for further study to draw stronger conclusions about the advantages of the use of acupuncture to treat children with ASD.

## Figures and Tables

**Table 1:** Evidence quality classification.

Certainty assessment							No. of patients		Effect		Certainty	Importance
No. of studies	Study design	Risk of bias	Inconsistency	Indirectness	Imprecision	Other considerations	Acupuncture	Rehabilitation training	Relative (95% CI)	Absolute (95% CI)		
*CARS*												
9	Randomised trials	Serious^a^	Serious^b^	Not serious	Not serious	None	356	367	—	MD 4.6 lower (5.09 lower to 4.1 lower)	⊕⊕○○ Low	Critical

*CARS, acupuncture subgroup*												
7	Randomised trials	Serious^a^	Not serious	Not serious	Not serious	None^c^	258	269	—	MD 2.65 lower (3.22 lower to 2.07 lower)	⊕⊕⊕○ Moderate	Critical

*CARS, acupuncture plus massage subgroup*												
2	Randomised trials	Serious^a^	Not serious	Not serious	Serious^d^	Publication bias strongly suspected^c^	98	98	—	MD 10.35 lower (11.34 lower to 9.36 lower)	⊕○○○ Very low	Critical

*ABC*												
10	Randomised trials	Serious^a^	Serious^b^	Not serious	Not serious	Publication bias strongly suspected^e^	411	408	—	MD 7.38 more (8.03 more to 6.74 more)	⊕○○○ Very low	Critical

*ABC (sensory)*												
2	Randomised trials	Serious^a^	Not serious	Not serious	Serious^d^	Publication bias strongly suspected^c^	76	75	—	MD 2.67 more (2.9 more to 2.44 more)	⊕○○○ Very low	Important

*ABC (social/self-help)*												
2	Randomised trials	Serious^a^	Not serious	Not serious	Serious^d^	Publication bias strongly suspected^c^	76	75	—	MD 2.09 more (2.3 more to 1.89 more)	⊕○○○ Very low	Important

*ABC (body and object use)*												
2	Randomised trials	Serious^a^	Not serious	Not serious	Serious^d^	Publication bias strongly suspected^c^	76	75	—	MD 1.19 more (1.38 more to 1 more)	⊕○○○ Very low	Important

*ABC (relating)*												
2	Randomised trials	Serious^a^	Not ious	Not serious	Serious^d^	Publication bias strongly suspected^c^	76	75	—	MD 3.28 more (3.55 more to 3.02 more)	⊕○○○ Very low	Important

*ABC (language)*												
2	Randomised trials	Serious^a^	Not serious	Not serious	Serious^d^	Publication bias strongly suspected^c^	76	75	—	MD 2.45 more (2.73 more to 2.16 more)	⊕○○○ Very low	Important

*RDLS (CY)*												
3	Randomised trials	Serious^a^	Not serious	Not serious	Serious^d^	Publication bias strongly suspected^c^	67	59	—	MD 0.15 higher (0.04 higher to 0.26 higher)	⊕○○○ Very low	Important
*RDLS (EY)*												
3	Randomised trials	Serious^a^	Not serious	Not serious	Serious^d^	Publication bias strongly suspected^c^	67	59	—	MD 0.08 higher (0.06 higher to 0.22 higher)	⊕○○○ Very low	Important

*WeeFIM*												
3	Randomised trials	Serious^a^	Not serious	Not serious	Serious^d^	Publication bias strongly suspected^c^	67	59	—	MD 3.7 higher (2.38 higher to 5.02 higher)	⊕○○○ Very low	Important

CI: confidence interval; MD: mean difference. *Note.*^a^The included studies are biased in allocation concealment and blinding. ^b^The degree of overlap of the credible intervals of different studies is poor, and the I2 value of the combined results is larger. ^c^ The number of included studies is small, and they are positive, and there is a possibility of greater publication bias. ^d^The sample size does not meet the OIS standard. The form is completed on the GRADE PRO website: http://www.guidelinedevelopment.org/. The symbol “⊕” in the figure represents the level, and the more ⊕, the higher the level. ^e^Asymmetric funnel chart.

**Table 2:** Characteristics of the included studies.

Authors, years	Age (C; T)	Gender (M : F)	Sample (C/T)	Intervention (C)	Intervention (T)	Outcome indicators	Acupuncture points	Course of treatment
Wang, 2016 [23]	C (7.8 ± 3.5) T (7.5 ± 4.0)	C (27 : 22) T (28 : 21)	49/49	Rehabilitation training	C + acupuncture and tuina + music therapy	CARS; ABC	Shenting (GV 24), Benshen (GB 13), Sishencong (EX-HN1), Touwei (ST 8), Shangxing (GV 23), Naohu (GV 17), Fengchi (GB 20), Shenmen (HT 7), Neiguan (PC 6), Laogong (PC 8), Zhongwan (CV 12), Guanyuan (CV 4), and Qihai (CV 6)	Once/day

Wang, 2018 [20]	C (4.24 ± 2.16) T (4.21 ± 2.12)	C (15 : 30) T (16 : 29)	45/45	Rehabilitation training	C + scalp acupuncture	ABC	Shen Ting (GV 24), Sishencong (EX-HN1), and Benshen (GB13)	3 times/week, with a pause of 15 days after 10 times; 30 times in total

Tang and Yuan, 2020 [24]	C (2–9) T (22∼41)	C (17 : 18) T (16 : 19)	35/35	Structured education model training	C + acupuncture	CARS; subitems of ABC	Zhi tri-needles (Shenting (GV 24), Benshen (GB 13) ^*∗*^ 2), temporal tri-needles (the three acupuncture points are located 2 cuns above the tip of the ear and 1 cun on the left and right sides), Si Shen needles (four points located at 1.5 inches on the Baihui (GV 20)), brain tri-needles (Naohu (GV 17), Naokong (GB 19)), foot tri-needles (Zulinqi (GB 41), Neiting (ST 44), Taichong (LR 3)), hand tri-needles (Quchi (LI 11), Waiguan (TE 5), Hegu (LI 4)), tongue tri-needles (the three acupuncture points are located 1 cun above the Lianquan (CV 23), and 0.8 cuns on the left and right sides)	Once/day, no less than 3 times a week; 6 consecutive months

Zhang et al., 2019 [21]	C (2–5) T (2–5)	C (25 : 5) T (26 : 4)	30/30	Sensory integration training and exercise intervention	C + scalp acupuncture	CARS; ABC; subitems of ABC	Si Shen needles (four points located at 1.5 inches on the Baihui (GV 20)), brain tri-needles (Naohu (GV 17), Naokong (GB 19)), temporal tri-needles (the three acupuncture points are located 2 cuns above the tip of the ear and 1 cun on the left and right sides), Zhi tri-needles (Shenting (GV 24), Benshen (GB 13) ^*∗*^ 2), foot tri-needles (Zulinqi (GB 41), Neiting (ST 44), Taichong (LR 3)), hand tri-needles (Quchi (LI 11), Waiguan (TE 5), Hegu (LI 4))	Once/day for 6 consecutive months

Dang et al., 2020 [25]	C (3–6) T (3–6)	C (34 : 9) T (32 : 11)	43/43	Rehabilitation training	C + acupuncture	ABC; CARS	Baihui (GV 20), Sishen needles (four points located at 1.5 inches on the Baihui (GV 20)), temporal tri-needles (the three acupuncture points are located 2 cuns above the tip of the ear and 1 cun on the left and right sides), brain tri-needles (Naohu (GV 17), Naokong (GB 19)), Zhi tri-needles (Shenting (GV 24), Benshen (GB 13) ^*∗*^ 2), and Dingshen needles (Yingtang (GV 29), Yangbai (GB 14) ^*∗*^ 2)	Once/day, continuous treatment for 6 days, 1 day off; total treatment for 6 months

Zeng and Ou-yang, 2017 [26]	C (3–7.5) T (3.5–8)	C (19 : 8) T (18 : 9)	27/27	Rehabilitation training	C + acupuncture	CARS; ABC	Sishencong (EX-HN1), Shenting (GV 24), Benshen (GB 13), Yingtang (GV 29), Naohu (GV 17), Naokong (GB 19), language area 1, language zone 2, and language zone 3	Three times a week, 1 month is the course of treatment, 4 consecutive courses of treatment

He et al, 2020 [27]	C (4–8) T (3–8)	C (27 : 13) T (25 : 16)	40/41	Rehabilitation training	C + scalp acupuncture	Subitems of ABC	Sishencong (EX-HN1), Benshen (GB 13), Yingtang (GV 29), Naohu (GV 17), Naokong (GB 19), language area 1, language zone 2 and language zone 3	3 times a week, 1 month is the course of treatment, 4 consecutive courses of treatment

lv et al., 2018 [28]	C (3–9.4) T (3–10)	C (41 : 8) T (39 : 10)	49/49	Rehabilitation training	C + acupuncture and tuina	CARS; ABC	Sishencong (EX-HN1), Benshen (GB 13), Shenting (GV 24), Touwei (ST 8), Naohu (GV 17), Shenmen (HT 7), Neiguan (PC 6), and Laogong (PC 8)	Once/day, 5 days/week, 10 times is the course of treatment; 6 months is the total time of treatment

Zhao et al., 2020 [29]	C (2.5–8) T (2.3–7.8)	C (33 : 17) T (38 : 12)	50/50	Rehabilitation training and special education	C + TCM treatment (including acupuncture)	CRAS; ABC	Baihui (GV 20), Sishen needles (four points located at 1.5 inches on the Baihui (GV 20)), Dingshen needles (Yingtang (GV 29), Yangbai (GB 14) ^*∗*^ 2), Shenting (GV 24), Benshen (GB 13), 2/5 under the parietotemporal anterior slash, language Zone 2 and language Zone 3, Naohu (GV 17), Shenmen (HT 7), Neiguan (PC 6), Xuan Zhong (GB 39), and Yongquan (KI 1)	Once/day, 6 days/week; 3 months of treatment in total

Yuan et al., 2013 [30]	C (5 ± 3) T (5 ± 2)	C (22 : 8) T (23 : 7)	30/30	Rehabilitation training	C + scalp acupuncture	CARS	Zhi tri-needles (Shenting (GV 24), Benshen (GB 13) ^*∗*^ 2), Sishen needles (four points located at 1.5 inches on the Baihui (GV 20)), and brain tri-needles (Naohu (GV 17), Naokong (GB 19) ^*∗*^ 2)	Once/day, 6 days of treatment per week, 3 months as a course of treatment; 1 course in total

Liu, 2017 [31]	C (2–8) T (3–7)	C (22 : 8) T (23 : 7)	30/30	Rehabilitation training after acupuncture	Rehabilitation training with acupuncture	CARS	Sishen needles (four points located at 1.5 inches on the Baihui (GV 20)), Dingshen needles (Yingtang (GV 29), Yangbai (GB 14) ^*∗*^ 2), brain tri-needles (Naohu (GV 17), Naokong (GB 19)∗2), foot tri-needles (Zulinqi (GB 41), Neiting (ST 44), Taichong (LR 3)), and hand tri-needles (Quchi (LI 11), Waiguan (TE 5), Hegu (LI 4))	Once/day, 6 days/week; 3 months of treatment in total

Wong et al., 2010 [17]	C (9.56 ± 4.22) T (9.17 ± 4.12)	C (22 : 3) T (25 : 5)	25/30	Rehabilitation training + sham acupuncture	Rehabilitation training + electroacupuncture	Subitems of ABC; subitems of RDLS WeeFIM;	Sishencong (EX-HN1), Yingtang (GV 29), Shenmen (HT 7), Taichong (LR 3), ear naodian (AT 3), ear shenmen (TF 4), and San yinjiao (SP 6)	30 min for each time, once/day for 4 weeks

Kong et al., 2018 [32]	C (6.8 ± 0.9) T (6.5 ± 0.8)	C (19 : 11) T (17 : 13)	30/30	Rehabilitation training	C + acupuncture	CARS; ABC	Brain tri-needles (Naohu (GV 17), Naokong (GB 19) ^*∗*^ 2), Sishen needles (four points located at 1.5 inches on the Baihui (GV 20)), Zhi tri-needles (Shenting (GV 24), Benshen (GB 13) ^*∗*^ 2), temporal tri-needles (the three acupuncture points are located 2 cuns above the tip of the ear and 1 cun on the left and right sides), hand tri-needles (Quchi (LI 11), Waiguan (TE 5), Hegu (LI 4)), Shou-Zhi needles (Shenmen (HT 7), Neiguan (PC 6), Laogong (PC 8)), tongue tri-needles (the three acupuncture points are located 1 cun above the Lianquan (CV 23) and 0.8 cun on the left and right sides), foot tri-needles (Zulinqi (GB 41), Neiting (ST 44), Taichong (LR 3)), Fengchi (GB 20), and Yamen (GV 15)	Once/day, 30 min for each time; 4-month treatment in total

Wong et al., 2014 [18]	C (8.75 ± 4.62) T (10.17 ± 3.93)		9/12	Rehabilitation training	TAC (tongue acupuncture)	Subitems of RDLS, WeeFIM	(TAC #1 run Ze, TAC #2 Guan Zhu) and (TAC #3 Tian men, TAC#4 and TAC #5 Di You')	5 times a week for 8 weeks

Wong and Sun, 2010 [19]	C (6 ± 5.92) T (6.23 ± 5.67)		25/25	Sham TAC	TAC (tongue acupuncture)	RDLS, FIM	(TAC #1 run Ze, TAC #2 Guan Zhu) and (TAC #3 Tian men, TAC#4 and TAC #5 Di You')	5 times a week for 8 weeks

Li et al., 2017 [33]		C (30 : 15) T (28 : 17)	45/45	Rehabilitation training	C + acupuncture	ABC	Shuigou (GV26), Fengfu (GV 16), Chengjiang (CV 24), Jiache (ST 6), Shaoshang (LU 11), Daling (PC 7), Yinbai (SP 1), Haiquan (EX-HN11), Laogong (PC 8), Shenmai (BL 62), Shangxing (GV 23), Huiyin (CV 1), and Quchi (LI 11)	3 times a week, with a 20-day interval after 10 times; 30 treatments in total constituted a course of treatment. The patients were treated for 3 courses

## Data Availability

The datasets used and analyzed in the current study are included within this article.
